# Anti-inflammatory agents and monoHER protect against DOX-induced cardiotoxicity and accumulation of CML in mice

**DOI:** 10.1038/sj.bjc.6603640

**Published:** 2007-02-27

**Authors:** A M E Bruynzeel, M A Abou El Hassan, C Schalkwijk, J Berkhof, A Bast, H W M Niessen, W J F van der Vijgh

**Affiliations:** 1 Department of Medical Oncology, VU University Medical Center, 1081 HV Amsterdam, the Netherlands; 2 Cancer Biology Department, National Cancer Institute, Cairo, Egypt; 3 Department of Internal Medicine, University of Maastricht, 6200 MD Maastricht, the Netherlands; 4 Department of Clinical Epidemiology and Biostatistics, VU University Medical Center, 1081 HV Amsterdam, the Netherlands; 5 Department of Pharmacology and Toxicology, Faculty of Medicine, University of Maastricht, 6200 MD Maastricht, the Netherlands; 6 Department of Pathology, VU University Medical Center, 1081 HV Amsterdam, the Netherlands; 7 ICaR-VU, VU University Medical Center, 1081 HV Amsterdam, the Netherlands; 8 Department of Cardiac Surgery, VU University Medical Center, 1081 HV Amsterdam, the Netherlands

**Keywords:** doxorubicin, cardiotoxicity, inflammation, N^*ε*^-(carboxymethyl)lysine, monoHER, anti-inflammatory agents

## Abstract

Cardiac damage is the major limiting factor for the clinical use of doxorubicin (DOX). Preclinical studies indicate that inflammatory effects may be involved in DOX-induced cardiotoxicity. N^*ε*^-(carboxymethyl) lysine (CML) is suggested to be generated subsequent to oxidative stress, including inflammation. Therefore, the aim of this study was to investigate whether CML increased in the heart after DOX and whether anti-inflammatory agents reduced this effect in addition to their possible protection on DOX-induced cardiotoxicity. These effects were compared with those of the potential cardioprotector 7-monohydroxyethylrutoside (monoHER).

BALB/c mice were treated with saline, DOX alone or DOX preceded by ketoprofen (KP), dexamethasone (DEX) or monoHER. Cardiac damage was evaluated according to Billingham. N^*ε*^-(carboxymethyl) lysine was quantified immunohistochemically.

Compared to saline, a 21.6-fold increase of damaged cardiomyocytes was observed in mice treated with DOX (*P*<0.001). Addition of KP, DEX or monoHER before DOX significantly reduced the mean ratio of abnormal cardiomyocytes in comparison to mice treated with DOX alone (*P⩽*0.02). In addition, DOX induced a significant increase in the number of CML-stained intramyocardial vessels per mm^2^ (*P*=0.001) and also in the intensity of CML staining (*P*=0.001) compared with the saline-treated group. N^*ε*^-(carboxymethyl) lysine positivity was significantly reduced (P⩽0.01) by DOX-DEX, DOX-KP and DOX-monoHER. These results confirm that inflammation plays a role in DOX-induced cardiotoxicity, which is strengthened by the observed DOX-induced accumulation of CML, which can be reduced by anti-inflammatory agents and monoHER.

Doxorubicin (DOX) is a successfully used anticancer drug. However, DOX-induced cumulative cardiotoxic effects, including cardiomyopathy and congestive heart failure, limit the use of this agent (Von Hoff *et al*, 1979; Signal and Iliskovic, 1998; Gharib and Burnett, 2002). Various molecular mechanisms have been suggested. Doxorubicin-induced free radicals are believed to play a central role in its cardiotoxicity (Yen *et al*, 1996; Horenstein *et al*, 2000; Xu *et al*, 2001).

Earlier studies illustrated that DOX also induces inflammatory effects in the vasculature and in the myocardium (Hecker, 1990; Fujihira *et al*, 1993) and increases proinflammatory cytokines (TNF-*α*, IL-1*β* and IL-2). Doxorubicin elevates NF-*κ*B (Baeuerle, 1991; Read *et al*, 1994; Goto *et al*, 1999; Hou *et al*, 2005; Deepa and Varalakshmi, 2006) and the adhesion molecules VCAM-1 and E-selectin (Abou El Hassan *et al*, 2003). *In vitro* data showed that DOX affected both the viability and neutrophil adhesion of endothelial cells with clinically achievable concentrations (Abou El Hassan *et al*, 2003). These inflammatory effects may play a role in DOX-induced cardiotoxicity and results of some studies support these indications (Inchiosa Jr and Smith, 1990; Chen *et al*, 2005; Hou *et al*, 2005).

Protein damage caused by oxidative stress, inflammation or hyperglycaemia leads to carbohydrate-derived advanced glycation end products (AGEs) such as N^*ε*^-(carboxymethyl)lysine (CML) (Miyata *et al*, 1997; Hudson *et al*, 2003). Elevated levels of CML were demonstrated in patients with renal failure, in intramyocardial arteries of the heart of diabetic patients (Schalkwijk *et al*, 2004) and in patients with atherosclerosis having inflammatory/pro-oxidative environments (Degenhardt *et al*, 1997; Schleicher *et al*, 1997). N^*ε*^-(carboxymethyl) lysine is produced under oxidative stress (Miyata *et al*, 1997; Nagai *et al*, 1997) and may therefore be regarded as a biomarker for local endogenous oxidative stress, next to local inflammatory stress (Baynes, 1991; Nerlich and Schleicher, 1999). After binding to the receptor for AGE, CML activates endothelial cells as indicated by the induction of adhesion molecules such as VCAM-1 (Boulanger *et al*, 2002). Therefore, the first aim of our study was to investigate whether CML increases in intramyocardial arteries after treatment with DOX. Because inflammatory processes are involved the second aim of our study was to investigate whether anti-inflammatory agents would reduce DOX-induced CML increase.

In the past, we have shown the cardioprotective properties of the antioxidant 7-monohydroxyethylrutoside (monoHER) against DOX-induced cardiotoxicity in mouse (Van Acker *et al*, 1997; Van Acker *et al*, 2000). *In vitro*, we have also shown that monoHER protects against DOX-induced inflammatory effects (Abou El Hassan *et al*, 2003). Therefore, the effect of monoHER on DOX-induced CML increase was also investigated in the *in vivo* mouse model.

Furthermore, a possible protective effect of the anti-inflammatory drugs ketoprofen (KP) and dexamethasone (DEX) on DOX-induced cardiotoxicity in comparison to the protective effect of monoHER was investigated in this model.

## MATERIALS AND METHODS

### Chemicals

7-Monohydroxyethylrutoside was kindly provided by Novartis Consumer Health (Nyon, Switzerland). The drug was formulated and dissolved as described before, giving a final concentration of 33 mg/ml (Bruynzeel *et al*, 2006). Formulated DEX (dexamethasone 4 mg/ml) was obtained from the Pharmacy Department, VU Medical Center (Amsterdam, the Netherlands). Before injection, the content of the ampoule was diluted in sterile saline to obtain a concentration of 2 mg/ml. Formulated KP (1% ketoprofen) was obtained from Merial B.V. (Amstelveen, the Netherlands). A volume of 0.5 ml KP was added to 19.5 ml PBS to obtain a concentration of 0.025% KP (0.25 mg/ml). Formulated DOX (doxorubicin hydrochloride, 2 mg/ml) was obtained from Pharmachemie B.V. (Haarlem, the Netherlands). Before injection, the content of the vial was diluted in a sterile 0.9% NaCl solution to a concentration of 1 mg/ml.

### Animals

Thirty-six male BALB/c mice (20–25 g) obtained from Harlan Nederland (Horst, the Netherlands) were kept in a light and temperature-controlled room (21–22°C; humidity 60–65%). The animals were fed a standard diet (Harlan Teklad) and allowed to eat and drink tap water *ad libitum*. The animals were allowed to adapt to the laboratory housing conditions for 2 weeks before starting the experiment.

### Experimental design

The protocol was approved by the ethics committee for animal experiments of the Vrije Universiteit (Amsterdam, the Netherlands) and the methodology was also in compliance with the UKCCCR guidelines on ethical use of animals.

Thirty mice were submitted to one of the following weekly dosing schedules for 6 weeks:

Group 1 (*n*=6): 0.1 ml 0.9% NaCl solution i.v.+0.3 ml 0.9% NaCl solution s.c. 60 min before i.v. injection, and 6, 24 and 48 h after i.v. injection
Group 2 (*n*=6): 4 mg/kg DOX i.v.+0.3 ml 0.9% NaCl solution s.c. 60 min before DOX and 6, 24 and 48 h after DOX
Group 3 (*n*=6): 4 mg/kg DOX i.v.+2 mg/kg KP s.c. 30 min before DOX, and 6, 24 and 48 h after DOX
Group 4 (*n*=6): 4 mg/kg DOX i.v.+8 mg/kg DEX s.c. 60 min before DOX, and 6, 24 and 48 h after DOX
Group 5 (*n*=6): 4 mg/kg DOX i.v.+500 mg/kg monoHER i.p. 60 min before DOX

DOX was administered via the tail vein. Six mice were killed just before starting treatment (control group) and their heart tissue was used as a control at the beginning.

During treatment and a 2-week observation period thereafter, body weight was determined twice a week as a measure of general toxicity. After the treatments and the observation period, the mice were killed.

### Tissue samples

The hearts were excised and the central part of both ventricles was cut into 5-mm-thick pieces of 2–3 mm, which were fixed in 2% phosphate-buffered glutaraldehyde solution or in 4% formalin.

### Histological analyses

After fixation in 2% phosphate-buffered glutaraldehyde solution the heart tissue was post-fixed in 1% osmium tetroxide. The tissue was then dehydrated through a graded series of ethanol solutions of 70–95% and embedded in JB-4 Plus resin. Thereafter 0.5–3.0-*μ*m-thick sections were cut with a glass knife. These semithin sections were examined by light microscopy and DOX-induced cardiac damage was evaluated according to Billingham *et al* (1978). For this purpose the percentage of cardiac cells that had been damaged was established. Cardiac myocytes with more than two vacuoles or loss of myofibrils were counted as deviant. The scoring area was measured using a commercially available interactive video overlay-based measuring system (Q-Prodit, Leica, Cambridge, UK; Vermeulen *et al*, 2001). For each mouse the number of aberrant myocytes per mm^2^ was scored.

### Immunohistochemical methods

After fixation in 4% formalin the heart tissue was embedded in paraffin. Paraffin-embedded cardiac tissue sections (4 *μ*m) were mounted on microscope slides and were deparaffinised for 10 min in xylene at room temperature and dehydrated by decreasing concentrations of ethanol. Sections were then stained with haematoxylin and eosin. Subsequent to deparaffinisation and dehydration, sections were incubated with 0.3% hydrogen peroxide in methanol for 30 min to block endogenous peroxidase activity. Sections were not heated to prevent artificial induction of CML by this procedure (Dunn *et al*, 1989). Sections were preincubated with normal rabbit serum (1:50, Dako, Glostrup, Denmark) for 10 min and incubated for 60 min with anti-CML (1 : 500), both at room temperature. After washing in phosphate-buffered saline (PBS), pH 7.4, sections were incubated for 30 min with rabbit anti-mouse biotin-labelled antibody (1 : 500, Dako) at room temperature and subsequently washed in PBS. After incubation with streptavidin horseradish peroxidase (1 : 200, Dako) for 60 min at room temperature, peroxidase was visualised with 3,3-diamino-benzidine-tetrahydrochloride/H_2_O_2_ (Sigma Chemical Company, St Louis, MO, USA) for 3–5 min.

The CML staining intensity was scored in the intramyocardial arteries. For the intensity scoring each positive vessel was given a score of: 1=weak positivity, 2=moderate positivity or 3=strong positivity, according to a previous study (Schalkwijk *et al*, 2004). Subsequently, the scoring area was calculated as described before (Vermeulen *et al*, 2001). For each mouse the total number of CML staining arteries per mm^2^ was scored. Thereafter the difference in the CML staining intensity of the intramyocardial arteries per mm^2^ was investigated between the experimental groups.

### Statistical analysis

For the analyses, the number of aberrant cardiac myocytes was log-transformed, yielding an unskewed variable. Differences between experimental groups were assessed using Student's two-sided *t*-test. The level of significance was set at 5%. Ninety-five percent confidence intervals (CI) on the original scale were obtained by exponentiating the upper and lower bounds of the 95% confidence intervals constructed on the log-scale. All calculations were performed with SPSS version 9.0 (SPSS, Chicago, IL, USA). For the analyses, the difference between the experimental groups regarding the number of vessels positive for CML staining and the intensity scoring per mm^2^ was assessed using Student's two-sided *t*-test. The level of significance was chosen at 5%. These calculations were also performed with SPSS version 9.0. To examine whether the contribution of moderately and strongly stained CML vessel walls differed among treatment groups, Fisher's exact test was applied and also Student's two-sided *t*-test.

## RESULTS

Animals appeared lively throughout the study and no behavioral changes were observed between the treatment groups. There were no signs of decreased activity, indicating low general toxicity. No significant differences were observed in weight between the experimental groups. No signs of gastrointestinal toxicity were observed in the mice treated with KP.

### Histological examination of the cardiomyocytes

Histology of the hearts from the control and saline group did not show damaged cardiac myocytes, indicating that environmental factors and treatment with saline did not influence cardiac health of the animals. Treatment with DOX alone induced a significant 21.6-fold (95% CI 6.2–74.5) increase of damaged cardiac myocytes in comparison to the saline-treated group (*P*<0.001). Heart tissue of all mice treated with DOX alone or in combination with KP, DEX or monoHER, particularly showed vacuolar degeneration, whereas loss of myofibrils was rarely detected.

Table 1 shows the ratio of the mean number of aberrant cardiac myocytes per mm^2^ in all groups in comparison to the group treated with DOX. The addition of KP 30 min before and 6, 24 and 48 h after DOX injection resulted in a significant protective effect by reducing the ratio of the mean number of abnormal cardiac cells per mm^2^ with a factor 4.4 (95% CI 1.4–14.3, *P*=0.021). When DEX was added 60 min before DOX injection and 6, 24 and 48 h after DOX administration, a significant protective effect was also detected (*P*=0.006). Cotreatment with DEX led to a 6.2-fold reduction of deviant cardiac cells (95% CI 1.9–20.0) compared to the mice treated with DOX alone. The protective effect by adding monoHER before DOX led to a significant 8.6-fold (*P*=0.002, 95% CI 2.6–27.8) reduction of abnormal cardiomyocytes.

Table 1 also shows the ratio of the mean number of aberrant cardiac myocytes per mm^2^ in treated versus saline treated animals. When KP or DEX was added before DOX administration, significantly more abnormal cardiac myocytes were observed in comparison to the saline group, indicating that the protection was not complete (for KP a 4.9-fold increase, 95% CI 1.4–17.0, *P*=0.014; for DEX a 3.5-fold increase, 95% CI 1.0–12.0, *P*=0.049). When monoHER was added before DOX treatment, no significant increase of aberrant cardiac myocytes was detected compared with the saline-treated group (*P*=0.137). No significant difference was found between the groups treated with the combinations DOX-monoHER, DOX-KP and DOX-DEX (P>0.05).

### Immunohistochemical staining of CML

**Figure 1:**
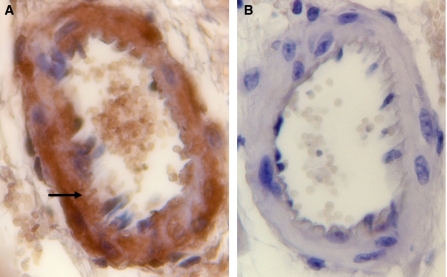
Immunohistochemical detection of CML in the mouse (× 63). Arrow: CML deposition on endothelial cells in intramyocardial blood vessel. (**A**) immunohistochemical detection of CML in the heart tissue of a mouse after DOX treatment alone, whereas (**B**) is an image without addition of the primary antibody.

**Figure 2:**
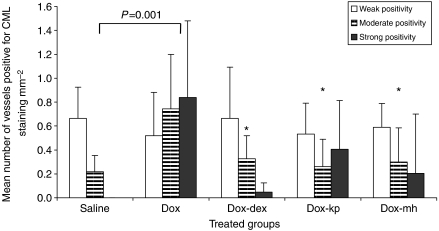
The mean number of vessels per mm^2^ weakly, moderately and strongly positive after staining for CML in intramyocardial vessels in the heart tissue of treated mice. A significant difference (*P*=0.001) was found between the mice treated with DOX alone and the animals treated with saline when the mean number of moderately plus strongly stained vessels per mm^2^ were considered (^*^ no significant difference in the mean number of strongly plus moderately stained CML vessels per mm^2^ in comparison to the saline-treated group and *P⩽*0.01 when compared with the DOX-treated group).

N^*ε*^-(carboxymethyl)lysine positivity was found in intramyocardial blood vessels, especially endothelium and partly smooth muscle cells in DOX-treated mice. Doxorubicin treatment induced a significant increase in the number of CML-stained vessels per mm^2^ compared with the group treated with saline (*P*=0.001) irrespective of the intensity score. Figure 1A illustrates immunohistochemical detection of CML in heart tissue of a mouse after treatment with DOX alone, whereas Figure 1B is a slide without addition of the primary antibody. Treatment of the animals with DOX in combination with DEX, KP or monoHER significantly reduced the amount of blood vessels positive for CML compared with the DOX-treated animals (*P*=0.004, 0.009 and 0.006, respectively). No difference was found in the number of vessels positive for CML between the groups treated with DOX combined with DEX, KP or monoHER and the animals treated with saline (*P*=0.633, 0.424 and 0.514, respectively). When comparing the amount of vessels positive for one of the three categories of intensity scores for CML (weak, moderate and strong) no difference was found between the five treatment groups for weakly stained positive CML vessels per mm^2^ (*P*=0.887), but when the mean number of moderately and strongly stained vessels per mm^2^ were combined for each experimental group, a significantly enhanced staining for CML (*P*=0.001) was found between the mice treated with DOX alone and the animals treated with saline (Figure 2). Dexamethasone, KP and monoHER reduced this enhancing effect of DOX significantly (*P*=0.003, 0.014 and 0.007, respectively). No significant difference in staining was found between the animals treated with saline and those treated with the combination DOX-DEX, DOX-KP or DOX-monoHER (*P*=0.659, 0.275 and 0.424, respectively). These results indicate that all three combinations significantly reduce the enhancing effect of DOX on CML intensity.

## DISCUSSION

In this study, we showed that addition of ketoprofen and dexamethasone during treatment with DOX reduced its cardiac damage *in vivo*. In addition, it was demonstrated that treatment with DOX induces an increase of CML in intramyocardial arteries in mice, which is reduced by these anti-inflammatory agents and monoHER.

Although DOX-induced free radicals are believed to play a central role in its cardiotoxicity (Yen *et al*, 1996; Horenstein *et al*, 2000, 57; Xu *et al*, 2001), the precise mechanism of myocardial impairment remains unclear. Several studies showed that inflammatory effects are directly and indirectly caused by treatment with DOX. *In vitro* it was shown that DOX directly induced neutrophil adhesion of vascular endothelial cells via the overexpression of VCAM and E-selectin (Abou El Hassan *et al*, 2003), whereas results of another study suggest that treatment with DOX produced marked inflammatory changes in heart tissue, liver and kidneys (Deepa and Varalakshmi, 2005). Results of our study confirm the contribution of inflammation in DOX-induced cardiotoxicity, because anti-inflammatory agents can at least, in part, reduce DOX-induced cardiotoxicity.

It has been suggested that DOX also induces endothelial dysfunctions (Kotamraju *et al*, 2002; Wolf and Baynes, 2006), because it has been demonstrated *in vivo* that treatment with DOX caused oxidative stress and myeloperoxidase (MPO) activity (Fadillioglu *et al*, 2004). N^*ε*^-(carboxymethyl)lysine can be formed by oxidative stress (Baynes, 1991; Nerlich and Schleicher, 1999), and also by the enzyme MPO (Anderson *et al*, 1999). In a recent study, was found that CML positivity colocalised with E-selectin-positive endothelial cells in the heart (Baidoshvili *et al*, 2006). Earlier it was demonstrated that DOX induced neutrophil adhesion that was mediated via overexpression of E-selectin (Abou El Hassan *et al*, 2003*).* Therefore, it is tempting to speculate that CML is derived from these pathways and could play a role in DOX-induced vascular endothelial injury and subsequent cardiotoxicity.

It is known that CML interacts with cells through a specific receptor system for AGEs (RAGE) (Zill *et al*, 2001). Activation of RAGE by binding of CML is thought to lead to the nuclear translocation of NF-*κ*B (Sousa *et al*, 2000) and the activation of several secondary messenger systems that increase the production of proinflammatory cytokines and adhesion molecules (Boulanger *et al*, 2002). These events lead to progressing inflammation and a further increase of formation and accumulation of CML. Several approaches have been used to block the formation of AGE or the interaction of AGEs with RAGE to reduce complications (Brownlee *et al*, 1986; Panagiotopoulos *et al*, 1998; Bucciarelli *et al*, 2002). From these studies it appeared that reduction or even prevention of the formation of CML seems to be important to prevent endothelial dysfunction, and besides, this also reduces inflammation.

In line with this, we have demonstrated in another study increased accumulation of CML in intramyocardial arteries of diabetic patients and suggested that CML contributes to the increased risk of heart complications in diabetes mellitus (C Schalkwijk and HWM Niessen, unpublished observation).

In the present study, we showed that monoHER significantly reduced CML positivity and intensity of intramyocardial arteries. As monoHER has been shown to have radical scavenging properties (Haenen *et al*, 1993; Van Acker *et al*, 1993, 1997, 2000), this again points to a role of free radicals in CML production by DOX. We also found that anti-inflammatory agents decreased CML positivity and intensity in intramyocardial arteries (Figure 1). It has indeed been suggested that inflammation is another source of CML formation (Daugherty *et al*, 1994; Anderson and Heinecke, 2003). It has, however, to be noticed that glucocorticoids and NSAIDs also have antioxidant properties (Hamburger and Mc Cay, 1990; Kataoka *et al*, 1997; Ozmen, 2005; Chen *et al*, 2005; Yamada *et al*, 2006) besides their anti-inflammatory properties (Koehler *et al*, 1990; Masferrer and Seibert, 1994; Auphan *et al*, 1995; Scheinman *et al*, 1995; Morteau, 2000).

As a representative of the NSAIDs, we used KP because it is a strong non-selective COX-inhibitor and it is available for s.c. injection. Dexamethasone was chosen as a representative of the glucocorticosteroids, because of its known strong anti-inflammatory properties.

By using the earlier mentioned treatment schedules for KP and DEX, we intended to maintain the presence of the anti-inflammatory agents when DOX was administered and during the first 2 days thereafter, because the high peak levels of DOX during that period (Van der Vijgh *et al*, 1990) are considered of major importance in the development of DOX-induced cardiotoxicity (Von Hoff *et al*, 1979).

Up to the present, two studies reported protective effects of cotreatment with ibuprofen and glucocorticoids on DOX-induced cardiac damage. The first study (Inchiosa Jr and Smith, 1990) only evaluated survival, whereas the effect of glucocorticoids on DOX toxicity was only evaluated *in vitro* (Chen *et al*, 2005). At present, our study quantifies to what extent cardioprotection occurred in animals cotreated with DEX, KP and monoHER. It strongly confirms the role of inflammation in DOX-induced cardiotoxicity and indicates a possible way to protect (in part) against this toxicity.

High-dose DEX or prednisone is part of the DOX containing therapeutic treatment regimens in patients with aggressive non-Hodgkin's lymphoma or multiple myeloma (VAD, CHOP). Considering the results of our study, we reviewed data of these clinical studies concerning the cardiac consequences of the combined use of DOX and glucocorticoids for these patients and found out that up to the present little is known about the long-term effects on their cardiac tissue (Limat *et al*, 2003; Elbl *et al*, 2006). These clinical aspects merit further attention.

As mentioned earlier, it was believed that the cardioprotective effect of monoHER was mainly owing to its radical scavenging and iron-chelating properties; however, the results of the present study in combination with the *in vitro* study of Abou El Hassan *et al* (2003) indicate that monoHER also has anti-inflammatory properties.

Recently, anti-inflammatory activity was also shown for the flavonoids quercetin (Comalada *et al*, 2005), myricetin (Kang *et al*, 2005) and luteolin (Kim and Jobin, 2005).

A quantitative comparison between the three compounds (KP, DEX and monoHER) regarding their intrinsic anti-inflammatory and/or radical scavenging activities is not possible yet, because none of the doses nor the dosing regimes of the investigated protectors are optimised.

In conclusion, two anti-inflammatory agents of different classes, ketoprofen (NSAID) and dexamethasone (synthetic glucocorticoid) clearly protected against DOX-induced cardiotoxicity in mice by decreasing the number of abnormal cardiac myocytes. These results establish the suggestion that inflammatory effects owing to treatment with DOX are involved in the development of DOX-induced cardiotoxicity. The role of DOX-induced inflammation in the development of its cardiac damage is confirmed by the observation that DOX induced accumulation of CML in intramyocardial arteries, which is significantly reduced after treatment with DEX, KP and monoHER. Further investigations are warranted to develop anti-inflammatory agents as a protector against DOX-induced cardiotoxicity.

## Figures and Tables

**Table 1:** Ratios of the mean number of aberrant cardiac myocytes/mm^2^

**Treatment group (n=6 per group)**	**Fold increase (95% CI, P)**	**Fold reduction (95% CI, P)**
1. Saline	1 (reference)	21.6 (6.2–74.5, <0.001)
2. DOX	21.6 (6.2–74.5, <0.001)	1 (reference)
3. DOX+KP	4.9 (1.4–17.0, 0.014)	4.4 (1.4–14.3, 0.021)
4. DOX+DEX	3.5 (1.0–12.0, 0.049)	6.2 (1.9–20.0, 0.006)
5. DOX+MH	2.5 (0.73–8.7, 0.137)	8.6 (2.6–27.8, 0.002)

Abbreviations: DEX=dexamethasone: DOX=doxorubicin; KP=ketoprofen; MH=7-monohydroxyethylrutoside.

Fold increase=geometric mean number of abnormal cells in treated animals/geometric mean number of abnormal cells in mice treated with saline, fold reduction=geometric mean number of abnormal cells in DOX-treated animals/geometric mean number of abnormal cells in other treatment groups.
